# Calcifying Odontogenic Cyst in Posterior Maxilla: A Case Report

**DOI:** 10.30476/dentjods.2024.102636.2375

**Published:** 2025-03-01

**Authors:** Farhad Ghorbani, Mohammad Amin Amiri, Mohammad Mehdi Farahmand, Saeed Afzoun, Maryam Paknahad

**Affiliations:** 1 Dept. of Oral of Maxillofacial Surgery, School of Dentistry, Shiraz University of Medical Sciences, Shiraz, Iran; 2 Oral and Dental Disease Research Center, Shiraz University of Medical Sciences, Shiraz, Iran; 3 Student Research Committee, Shiraz University of Medical Sciences, Shiraz, Iran; 4 Oral and Dental Disease Research Center, Dept. of Oral of Maxillofacial Radiology, School of Dentistry, Shiraz University of Medical Sciences, Shiraz, Iran

**Keywords:** Calcifying odontogenic cysts, Odontogenic cysts, Odontogenic tumors, Fractal dimension, Fractal analysis

## Abstract

In the present study, we reported a 66-year-old woman with an uncommonly painful calcifying odontogenic cyst (COC) in the posterior region of the left side of the maxilla. The cyst was evaluated radiographically and histopathologically. The present case showed a multilocular cyst with a mixed internal structure. The most noticeable effects on the peripheral structures were elevated maxillary sinus floor, osteomeatal complex, and nasal obstruction. To better understand the impact of COC on the trabecular pattern of the surrounding bone, we performed fractal analysis on the panoramic images pre- and post-operatively. The expansion of COC can change the trabecular pattern, which subsequently can change the fractal dimension of the area. After histopathological confirmation of the diagnosis, the cyst was surgically removed.

## Introduction

Calcifying odontogenic cyst )COC(, also known as Gorlin cyst, is a rare type consisting of 0.3-0.8% of all odontogenic cysts [ 1 ]. This cyst is characterized by ameloblastoma-like epithelium with focal ghost cells [ 1 - 2 ]. This type of odontogenic cyst is usually recognized as a benign lesion with a fair prognosis; however, the clinical and histological behavior of COC can include cystic, solid (neoplastic), and aggressive (malignant) variants [ 3 - 7 ]. 

COC has been recognized as the type of developmental cyst in the 4th classification of WHO in 2017 [ 8 ]. This cyst is commonly reported in the anterior part of the jaws [ 9 - 10 ] in the second and third decades of the patient's life [ 11 ]. The treatment approach for COC is dependent on the type of lesion. For most of the cases that indicate a benign nature, a conservative treatment approach, such as enucleation or marsupialization, is indicated [ 9 , 12 ]; however, in solid masses and aggressive lesions, en bloc resection with long-term follow-ups is recommended [ 9 ]. Here, we report a case of COC in a 66 Y/O patient, which was an unusual age for COC, and the posterior part of the maxilla, which was an unusual location for it. Pain was also an uncommon clinical sign in COC.

## Case Presentation

**Figure 1:**
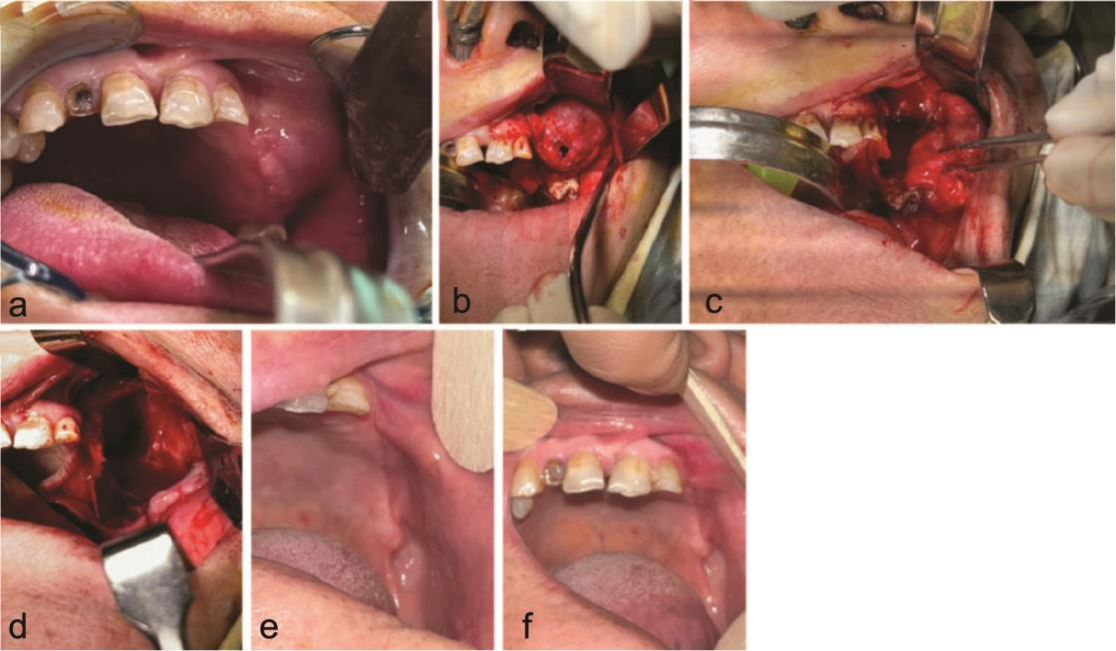
Intraoral view of the patient during, **a:** primary intraoral examination, **b:** surgical exposure of the cyst, **c:** surgical removal of the cyst, and **d:** after the surgical removal of the cyst, **e** and **f:** Intraoral view of the patient three months after surgery

A 66-year-old female patient with a history of an enlarged and painful lesion in the left part of the maxilla was admitted. A thorough medical history was taken from the patient. Due to her hypertension, she was taking amlodipine, aspirin, metoral, and atorvastatin. In the intra-oral examination, the lesion was hard in palpation and fluctuated in some areas (Figure 1). The vitality test (cold test) was positive for the first and second premolars, and the first molar in the left side of the maxilla.

### Radiographic Findings

**Figure 2:**
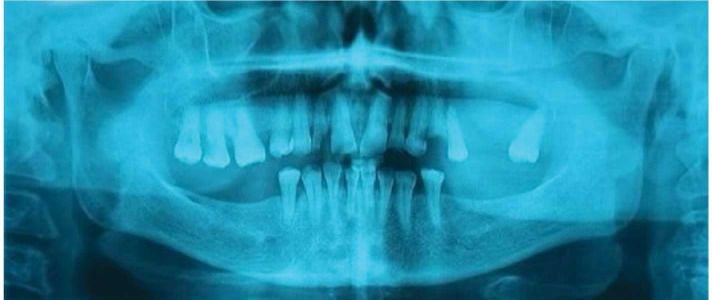
Panoramic view of the patient before surgery

**Figure 3:**
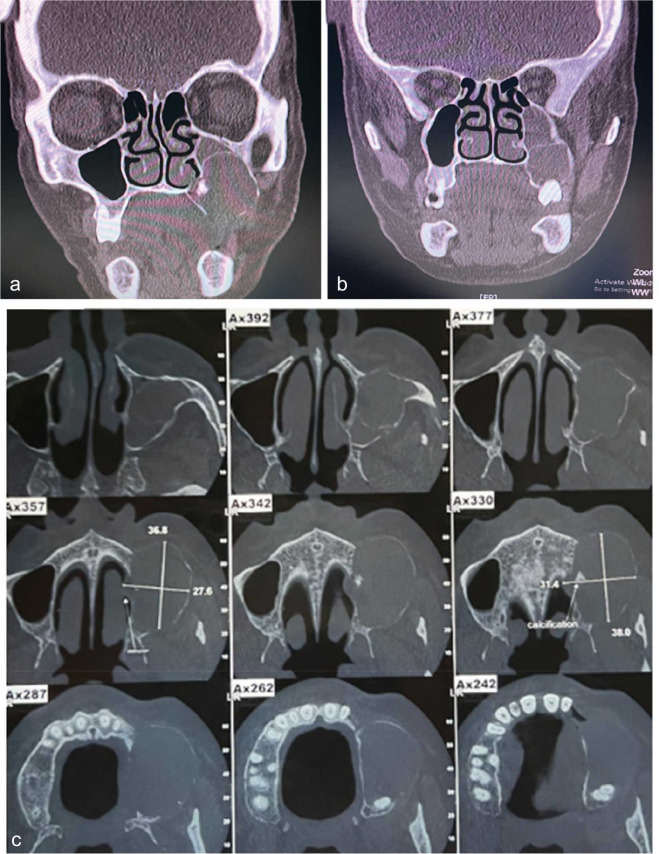
**a** and **b:** The coronal views and, **c:** cone beam computed tomography (CBCT) axial sections with measurement of the anteroposterior and mediolateral extensions of the cyst

A panoramic radiography and a cone-beam computed tomography (CBCT) scan were ordered for the patient (Figures 2-3). There was a well-defined corticated mixed lesion in the left side of the maxilla extending from the first premolar tooth to the third molar tooth. The lesion caused thinning and loss of continuity of the buccal and palatal cortices and the alveolar crest. Left nasal cavity obstruction, osteomeatal complex impotency, and mucosal thickening of the left maxillary sinus were detected. Elevation of the sinus floor and expansion of the sinus walls except the superior border was seen. Missing of the left maxillary first and second molar teeth was observed. The differential diagnosis was defined as long-standing cyst, cemento-ossifying fibroma, and COC.

### Fractal analysis of radiographic images

To assess the possible effects of the COC progression on the quality and the orientation of the surrounding bone trabeculae, fractal analysis of the trabecular pattern around the COC was undertaken.

### Image processing

**Figure 4:**
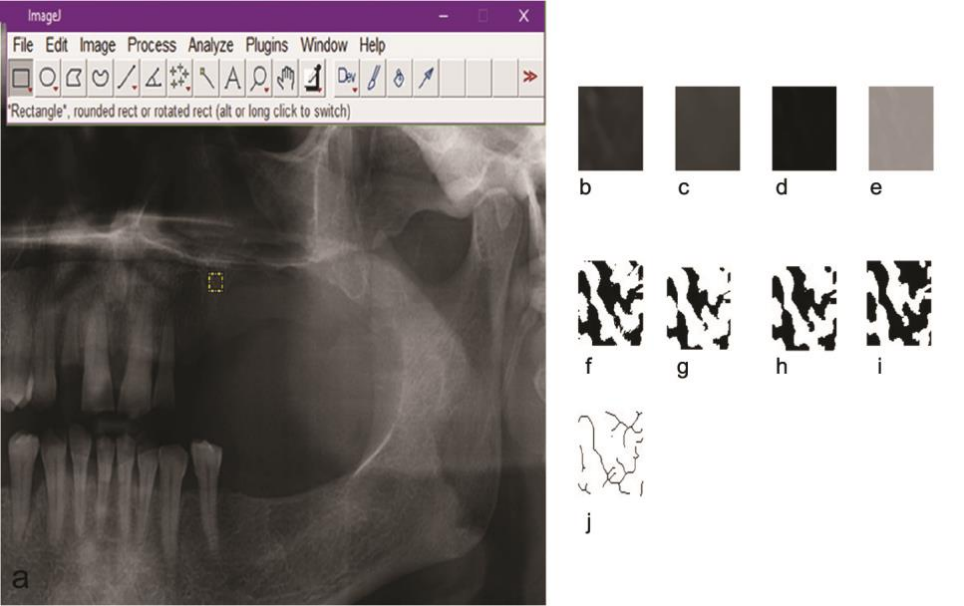
Selection of the region of interests (ROIs) by image J software. The image processing steps before the fractal analysis are represented as follows: image duplication, **b:** Addition of Gaussian blur filter, **c:** Subtraction from the original filter, **d:** Enhancement of image value to 128, **e:** Making the image binary, **f:** Eroded, **g:** Dilated, **h:** Inverted, **i:** and finally the images were skeletonized, **j:** to make them ready for fractal analysis

The panoramic radiographs taken before and 4 months after the surgery were chosen for the fractal analysis. In each of the radiographs, four regions of interest (ROI) with the size of 40 pixels 40 pixels were selected around the lesion as illustrated in Figure 4a. Using Image j software (https://imagej.nih.gov/il/), we processed the images to perform the fractal analysis. In the first step, the ROIs were duplicated, and a Gaussian blur filter was added to the image as shown in Figures 4b-c. In the next step, the images were subtracted from the original image, and 128 values were added to the images (Figures 4d-e, respectively). Subsequently, the image became binary, eroded, and dilated (Figures 4f- h). During the next step called inversion (Figure 4i), the trabeculae was made black before the skeletonization process. Eventually, the images were skeletonized (Figure 4j) to make them ready for the fractal analysis. 

### Fractal analysis

To perform the fractal analysis, the box-counting method was used in image J. The image was divided into boxes 2-64 pixels. The slope of the line of the logarithmic graph with the Y axis representing the number of boxes and the X axis representing the size of the boxes estimates the fractal dimension. After calculating the fractal dimension of four corresponding areas in the panoramic images before (1.25±0.055) and after (1.33±0.059) the surgery, the results indicated an enhanced level of fractal dimension.

### Treatment

### Surgical Procedure

**Figure 5:**
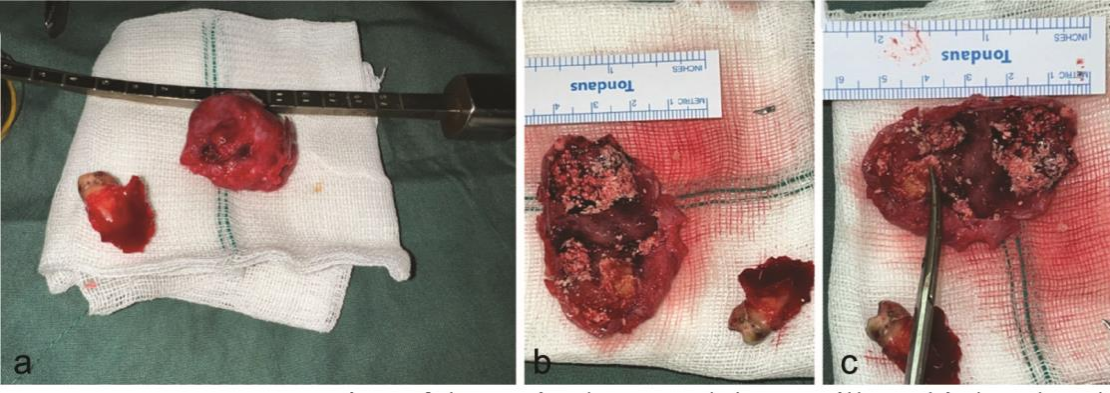
**a:** Representation of the excised cyst and the maxillary third molar along with the width, **b:** and the length, **c:** of the cyst

A biopsy was performed before the surgery, and the cyst was planned for surgical removal. To remove the cyst, we proposed surgical cyst enucleation for the patient. The patient underwent general anesthesia with special monitoring of the blood pressure. A crestal incision was made on the left side of the alveolar ridge of the maxilla. After the completion of the incision, the myomucosal flap was reflected to expose the cystic area. The cyst was totally excised from the area along with the third molar, which was engaged with the cyst, and a sample was obtained for further pathological evaluation (Figure 5). Moreover, curettage was performed in the marginal bone of the enucleate area. Then, the area was chemically cauterized using a hydrogen peroxide solution. The resultant maxillary bone defect was reconstructed with the “advancement of buccal fat”. After copious irrigation the flap was repositioned and sutured in two layers using Vicryl 3/0.

### Histopathological Findings

**Figure 6:**
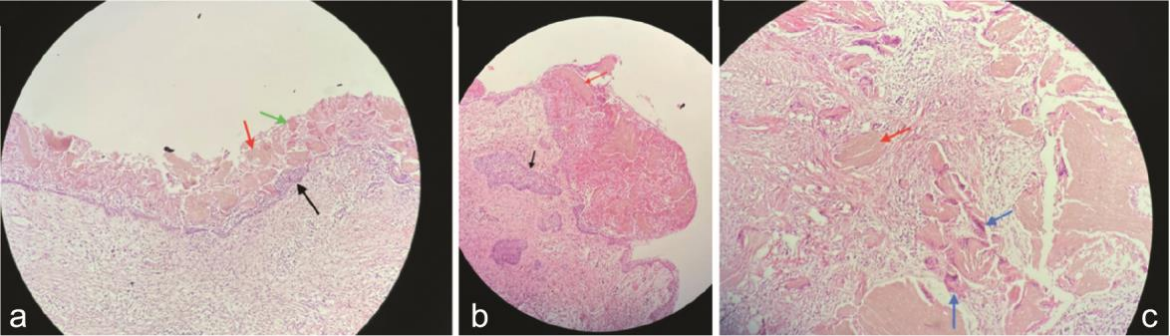
Histopathological section of the cystic epithelium (black arrow). The keratinized ghost cells are seen in the upper layers (red arrow) with some areas of dystrophic calcification (green arrow). **b:** The invasion of the epithelial nests into the connective tissue is represented (black arrow) with the large sheets of ghost cells (red arrow). **c:** In the case of the invasion of ghost cell nests (red arrow), the inflammatory reaction is seen as the aggregation of giant cells around the ghost cells (blue arrow)

The enucleated cyst was histopathologically evaluated. The histology sections indicated a cyst lined by odontogenic epithelium. The basal cells were palisaded and supporting a loosely arranged epithelium, which was undergoing ghost cell changes. The thick fibrous wall around the lesion consisted of multiple daughter cysts, foreign body reaction to herniated ghost cells, and dystrophic calcifications. Moreover, giant cells, as the signs of inflammation, are seen surrounding the ghost cells invading the connective tissue (Figure 6).

### Definitive diagnosis

Based on the evidence of the patient’s history, radiographical examinations, and clinical and histopathological results, the patient was diagnosed with COC.

### Follow-up Findings

**Figure 7:**
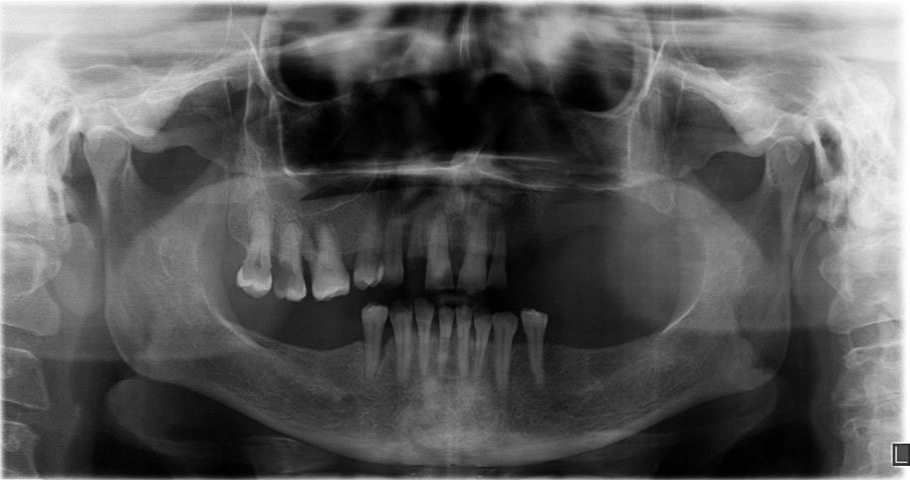
Panoramic view of the patient 3 months after the surgery. Note the left side of the maxilla

After a 10-day follow-up, the scars were sufficiently healed and the histopathological results confirmed the COC. Four months after the surgery, the patient was examined again, and a panoramic radiography was ordered to evaluate the healing area (Figure 7). 

## Discussion

COC is a rare tumor from the odontogenic epithelium. This benign cyst encompasses a wide range of histological and clinical variation [ 4 ]. Among all these variations, the most common manifestation of COC is reported to be cystic in 86-98% of the cases [ 4 ]. Since COC is developed from the trapped odontogenic epithelium in the jaw bones or the gingiva, it can also be classified as central (intraosseous) and peripheral (extraosseous) [ 13 ]. 

Concerning the common locations of COC in the oral cavity, 65-67.5% of the cysts are diagnosed in the anterior region of the jaws [ 4 ] with equal prevalence between the maxilla and the mandible [ 3 , 14 ]. More precisely, 75% of the cases are seen in the canine-incisor region [ 15 ]. Concerning the location of the cysts in both jaws, it is stated that the cysts in the mandible usually cross midline; nevertheless, the ones in the maxilla do not commonly represent the same pattern [ 16 - 17 ].

Regarding the clinical features of COC, some studi-es have indicated that these lesions are usually asymptomatic with bone expansion and jaw swelling [ 18 - 19 ]. 

The asymptomatic nature of COC results in its inci-dental discovery during radiography [ 7 ]. In radiographic examination of COC, it is stated that these lesions usually appear as unilocular with well-defined borders [ 1 ]. The multilocular COCs are reported in 5-13% of the cases [ 1 ]. The structure of the cyst encompasses a wide range from completely radiolucent to mixed radiolucent/radiopaque with uneven distribution of radiopacities [ 17 ].

In the present study, we have reported a case of COC with unique features including spontaneous pain in the area with no sign of superimposed infection, involvement of the left side of the maxilla extending from 1^st^ premolar region to the 3^rd^ molar, obstruction of the nasal cavity and osteomeatal complex, and eventually, the age of the patient (6^th^ decade of life). The rare findings of this case in the radiography evaluation are attributed to the location of the cyst. Since the reported COC was extended to the third molar region, the resultant expansion of the lesion could definitely affect the maxillary sinus, osteomeatal complex, and nasal cavity. Concerning the histopathological examination, the results represented a typical type of COC with sheets of ghost cells above the basal epithelial layer and the surrounding giant cells the ghost cell sheets that invaded the connective tissue layer. 

To have a more detailed understanding of the trabecular pattern of the bone surrounding the COC and its changes after the cyst enucleation, the authors of the current study used the fractal analysis technique. To the best of our knowledge, this study is the first one assessing the fractal dimension in a case of COC. The fractal analysis of radiographic images reveals the level of the trabecular complexity and the trabecular thickness, which is usually in the range of 1 to 2 in the osseous lesions [ 20 ]. Fractal analysis is a mathematical method by which irregular and complex body structures may be evaluated. The quantitative outcome of this method is defined as the fractal dimension. Since 1875, when du Bois Reimond first introduced the concept of continuous non-distinguishable functions, fractal analysis has been further improved and used by researchers [ 21 ]. In dentistry, assessment of the bone pattern of the jaws in dental radiographs is the main outcome evaluated using fractal analysis. The authors of the current study found that the bone surrounding the COC had a lower grade of fractal dimension than the healed bone after the surgery. This means the trabecular complexity, in terms of the trabecular orientation and the branches, is decreased when the COC expands and pushes the bone and its trabeculae away. The resultant expansion can reorient the trabeculae of the surrounding bone, since less space is left between the trabeculae to let them disperse in any orientation. The decreased fractal dimension is associated with decreased bone mineral density [ 22 ], which can be correlated with the mechanical strength of the bone [ 23 ]. This pattern may be useful in identifying the possible approach to cyst/tumor invasion since invasion is more frequently seen in the areas with less resistance to trabecular and bone resorption letting the cyst/tumor expand [ 17 ]. Further studies to evaluate the effect of other pathologic lesions and the healing process on the trabecular pattern and fractal dimension of the bone structure are recommended.

## Conclusion

COC is an uncommon benign odontogenic lesion with a higher frequency in the upper maxilla, which is most often asymptomatic. COC is a rare entity of odontogenic lesions but needs attention for correct diagnosis to avoid misdiagnosis and choosing the proper adequate treatment. The diagnosis of COC needs pathology confirmation due to the diverse clinical presentation and imaging features of odontogenic lesions to rule out the possibility of odontogenic tumors. The present study reported a case of COC in a 66-year-old patient, which was an unusual age for COC, and the posterior part of the maxilla, which was an unusual location for it. In addition, pain was an uncommon clinical sign in COC.

## References

[1] Ahmad SA, Popli DB, Sircar K, Hasan S ( 2022). Calcifying odontogenic cyst: Report of an uncommon entity with a brief literature review. J Oral Maxillofac Pathol (JOMFP)..

[2] De Arruda JAA, Monteiro JLGC, Abreu LG, de Oliveira Silva LV, Schuch LF, de Noronha MS, et al ( 2018). Calcifying odontogenic cyst, dentinogenic ghost cell tumor and ghost cell odontogenic carcinoma: A systematic review. J Oral Pathol Med.

[3] Sciubba JJ Tumors and cysts of the jaw. https://archive.org/details/tumorscystsofjaw0029sciu.

[4] Neville BW, Damm DD, Allen CM, Chi AC ( 2015). Oral and Maxillofacial Pathology.

[5] Hong SP, Ellis GL, Hartman KS ( 1991). Calcifying odontogenic cyst: A review of ninety-two cases with reevaluation of their nature as cysts or neoplasms, the nature of ghost cells and subclassification. Oral Surg Oral Med Oral Pathol.

[6] Toida M ( 1998). So‐called calcifying odontogenic cyst: Review and discussion on the terminology and classification. J Oral Pathol Med.

[7] Zornosa X, Müller S ( 2010). Calcifying cystic odontogenic tumor. Head Neck Pathol.

[8] Wright JM, Vered M ( 2017). Update from the 4th Edition of the World Health Organization Classification of Head and Neck Tumours: Odontogenic and Maxillofacial Bone Tumors. Head Neck Pathol.

[9] Thinakaran M, Sivakumar P, Ramalingam S, Jeddy N, Balaguhan S ( 2012). Calcifying ghost cell odontogenic cyst: A review on terminologies and classifications. J Oral Maxillofac Pathol (JOMFP)..

[10] Aristizabal Arboleda P, Sánchez-Romero C, de Al-meida OP, Flores Alvarado SA, Martínez Pedraza R ( 2018). Calcifying odontogenic cyst associated with dentigerous cyst in a 15-year-old girl. Int J Surg Pathol.

[11] Buchner A, Merrell PW, Carpenter WM, Leider AS ( 1990). Central (Intraosseous) calcifying odontogenic cyst. Int J Oral Maxillofac Surg.

[12] Lagarde X, Sturque J, Fenelon M, Marteau JM, Fricain JC, Catros S ( 2019). Calcifying odontogenic cyst: A report of two clinical cases. J Oral Med Oral Surg.

[13] Moradzadeh Khiavi M, Mahdavi N, Awudu A ( 2021). Developing odontoma arising from calcifying odontogenic cyst: a case report. Clin Case Reports.

[14] Buchner A ( 1991). The central (Intraosseous) calcifying odontogenic cyst: An analysis of 215 cases. J Oral Maxillofac Surg.

[15] Chrcanovic BR, Gomez RS ( 2016). Peripheral calcifying cystic odontogenic tumour and peripheral dentinogenic ghost cell tumour: An updated systematic review of 117 cases reported in the literature. Acta Odontol Scand.

[16] Chandran A, Nachiappan S, Selvakumar R, Gunturu S, Lakshmi UV, Bharathi K, et al ( 2021). Calcifying epithelial odontogenic cyst of maxilla: Report of a case and review and discussion on the terminology and classification. J Microsc Ultrastruct.

[17] White SC, Pharoah MJ ( 2014). Oral Radiology-E-Book: Principles and Interpretation.

[18] Saskianti T, Tedjosaongko U, Pramudita RA, Naomi N, Restu AR ( 2022). Multidisciplinary case management in mesiodens impacted cases with calcifying odontogenic cyst. Case Rep Dent.

[19] De Moraes ATL, Soares HA, Pinheiro J de JV, Ribeiro ALR ( 2020). Marsupialization before enucleation as a treatment strategy for a large calcifying odontogenic cyst: Case report. Int J Surg Case Rep.

[20] Kato CNAO, Barra SG, Tavares NPK, Amaral TMP, Brasileiro CB, Mesquita RA, Abreu LG ( 2020). Use of fractal analysis in dental images: A systematic review. Dentomaxillofacial Radiol.

[21] Mesquita RA ( 2020). Use of fractal analysis in dental images: a systematic review. Dentomaxillofac Radio.

[22] Alman AC, Johnson LR, Calverley DC, Grunwald GK, Lezotte DC, Hokanson JE ( 2012). Diagnostic capabilities of fractal dimension and mandibular cortical width to identify men and women with decreased bone mineral density. Osteoporos Int.

[23] Haba Y, Lindner T, Fritsche A, Schiebenhöfer AK, Souffrant R, Kluess D, et al ( 2012). Relationship between mechanical properties and bone mineral density of human femoral bone retrieved from patients with osteoarthritis. Open Orthop J.

